# Autoscribe: An automated tool for creating transcribed TextGrids from audio-recorded conversations

**DOI:** 10.3758/s13428-025-02850-9

**Published:** 2025-11-03

**Authors:** Tyson S. Barrett, Camille J. Wynn, Lotte Eijk, Katerina A. Tetzloff, Stephanie A. Borrie

**Affiliations:** 1 Department of Speech and Hearing Sciences, Utah State University, Logan, UT USA; 2 Department of Psychology, University of York, York, UK

**Keywords:** Conversation analysis, Speech transcription, Automation tool

## Abstract

One major difficulty in conversational research is the time required to segment and transcribe conversational recordings. While recent advances have improved automatic speech recognition technologies, one limitation of current tools is that they are generally catered toward speech that occurs in monologues rather than conversation. Accordingly, the purpose of this project was to develop and validate an automated user-friendly tool for transcribing conversations. This tool, called Autoscribe, converts dyadic conversational audio recordings into Praat TextGrids with time-aligned turn boundaries between speech and non-speech segments and transcripts of all spoken dialogue output. Here we describe the development of this tool as well as its validation on two conversational corpora. Results showed that Autoscribe decreased the amount of active working time needed for TextGrid creation by over 70%. Average transcription accuracy was 92% and average utterance boundary placement of 95%. Thus, Autoscribe affords a practical research tool that drastically reduces the time and resource intensitivity needed for conversational segmentation and transcription.

## Introduction

Technological advances continuously alter the way we perform research. Within the realm of communication research—spanning fields such as linguistics, psychology, business, and speech-language pathology—a surge of new tools have been developed to automate data coding and analysis tasks that were previously done by hand. For instance, natural language processing tools are used for tasks such as tagging parts of speech (e.g., Chiche & Yitagesu, 2022), topic segmentation (e.g., Arnold et al., 2019), or sentiment analysis (e.g., Wankhade et al., 2022). Other tools, such as Autoscore (Barrett et al., 2019; Borrie et al., 2019) and ALIGN (Duran et al., 2019), help transform linguistic data into quantitative scores that can be used in statistical analysis. The advantage of these tools lies in their efficiency and reproducibility. Tasks that are time-consuming and resource-intensive to complete by hand can be completed quickly at little to no cost and often can be fully reproduced. In some instances, tools are largely automatic, but, due to current technological limitations, still require human labor or oversight to complete certain tasks (e.g., Pfeifer et al., 2024). For instance, tools may require human checking and correcting to ensure higher accuracy (as is the case in the tool developed here). Even in these cases, the amount of data that can be analyzed and processed far exceeds what could be done by human power alone.

One tool that has been especially useful for research in both speech and language is automatic speech recognition (ASR) technology, which allows spoken language from an audio file to be transcribed into readable text. This technology allows for notable advances in linguistic research. For example, ASR has allowed researchers to scale investigations to much larger corpora and to conduct faster analyses of spoken language that would have been impractical with manual transcription alone. However, one of the major problems with ASR tools is that they are generally catered toward monologic speech (i.e., speech from one speaker; Lopez et al., 2022). Accordingly, though some tools are capable of transcribing conversational recordings, they are often ill-suited to interactional speech and language research. This necessitates the development of tools better equipped to manage the transcription and coding of conversations for subsequent analysis.

Adequate conversational tool development must focus on the key differences between monologues and conversations and how these differences can be managed most effectively. The most obvious way in which conversational speech differs from monologues is that conversation always involves at least two interlocutors. Thus, labels must clearly demarcate who is speaking and when. Next, conversations involve turn-taking. Interlocutors alternate between speaking and listening roles. Conversational turns need to be annotated (i.e., dividing segments of speech and non-speech for each), and analyses of phonetic and prosodic features of speech, as well as turn-taking dynamics (e.g., inter-turn latencies), require the time-alignment of these segments to be both accurate and precise. Further, while simplistic turn-taking models (and most automated tools currently used to transcribe speech) presume that only one interlocutor is speaking at a time, this is not always the case in real-world spoken dialogue. Rather, conversation is characterized by frequent interruptions, overlapping turns, and interjections (Brusco et al., 2020; Yuan et al., 2007). Therefore, simultaneous signals must be effectively managed. Finally, the linguistic conventions of conversation differ from those often used in monologues. Conversations contain back-channels (e.g., mmhmm, uh-huh), in addition to more disfluencies, filler words, and colloquialisms than monologues (e.g., Fox Tree, 1999). Thus, because ASR tools are often trained on speech in monologues (e.g., Ardila et al., 2020; Panayotov et al., 2015), they fail to capture these types of words. In fact, some tools capitalize on readability by intentionally filtering out these elements (e.g., Chen et al., 2022; Jamshid Lou & Johnson, 2020). However, these types of words are often intentionally used by speakers for a variety of purposes (e.g., signaling engagement, promoting conversational flow, expressing uncertainty, managing discourse) that necessitate their inclusion in many types of analyses (e.g., Clark & Fox Tree, 2002; Knudsen et al., 2020).

With these factors in mind, it becomes important to consider the format in which an automated tool could best code conversational data while ensuring that the complexity and depth of information is effectively handled. One format that is especially effective is a Praat TextGrid. Praat is an accessible and open-source software that offers a number of features for linguistic analysis (Boersma & Weenink, 2024). This tool is used extensively in speech and language research. According to Google Scholar, it has been cited over 32,000 times. Germane to this paper is the TextGrid function, which can be used to segment, annotate, and transcribe conversational data. Praat TextGrids allow for the creation of multiple tiers, each containing different pieces of information.

Thus, conversational data from each interlocutor can be annotated onto a separate tier, allowing for a straightforward delineation of multiple (and often simultaneously speaking) interlocutors. Within each interlocutor’s tier, boundaries can be added to delineate speech and non-speech segments, and speech segments can be transcribed. This allows for precise information regarding the timing of conversational turns, which can be important for analyses of conversations (e.g., when exploring inter-turn latencies). Further, because each interlocutor’s transcripts are contained on different tiers, overlapping speech signals are managed more effectively than can be captured in a simple linear transcript of a conversation. Praat TextGrids allow the integration of audio, audio visualizations (i.e., audio waveforms and spectrograms), and linguistic annotations into one place, allowing the conversational data to be used for a variety of different analyses across both speech and language domains. As conversation involves the interplay and coordination of multiple levels of language (e.g., phonetic, syntactic, and semantic) in tandem, this integration of information is especially advantageous for research in this area. Therefore, Praat TextGrids offer a functional and versatile format for conversation data that can be used for many different types of analyses.

While Praat TextGrids are notable for their practicality and usefulness, they are tedious and time-consuming to create manually. In our labs’ experiences, this process, which includes creating a TextGrid shell with the correct parameters, annotating boundaries between speech and non-speech segments, and transcribing speech, takes an estimated average of 15 minutes for every minute of conversational speech. While there are tools that automate single steps within this process, there are few automated tools with the capability of completing the entire TextGrid creation process. Liu and colleagues (2023) created an automated transcription tool with the capability of converting transcripts into TextGrids and showed that this tool works efficiently with a high degree of accuracy. However, the transcription format of this tool (i.e., Codes for the Human Analysis of Talk [CHAT] transcription format; MacWhinney, 2000), contains many features that are not conducive to certain types of analyses. For instance, utterances are divided by C-units (i.e., main clauses with associated dependent clauses) rather than interpausal units (pause-free units of speech), making it impossible to analyze turn boundaries. Additionally, this tool is a command-line program, making it inaccessible to those without at least a basic understanding of computer coding. Ma and colleagues (2024) also created a similar tool. However, this tool is not free or open-source and does not ensure participant confidentiality (i.e., audio recordings are sent to a third-party).

**Fig. 1:**
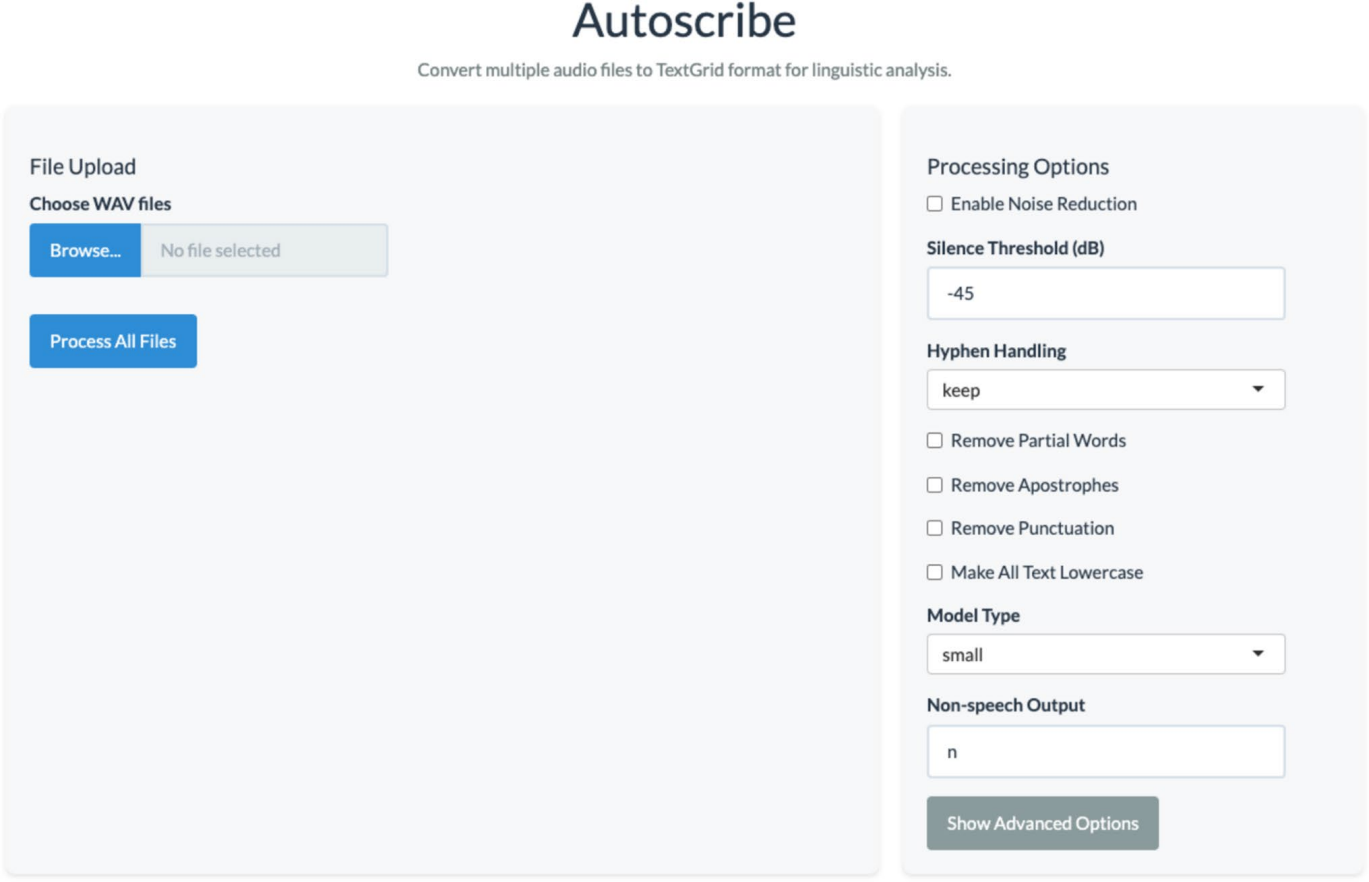
Screenshot of Autoscribe desktop application

Accordingly, the purpose of this project was to develop and validate an automated user-friendly tool (available as both a desktop application and an R package) for conversational segmentation and transcription. A screenshot of the desktop application can be viewed in Fig. 1. This tool, Autoscribe, converts dyadic conversational audio recordings (with each interlocutor recorded on a separate channel) into Praat TextGrids with time-aligned turn boundaries between speech and non-speech segments and transcripts of all spoken dialogue output. Given the limitations of automatic speech recognition and boundary alignment tools, our goal here was not to fully eliminate the need for human oversight in the TextGrid creation process. Rather, we aimed to limit the involvement of human coders to checking and correcting automatically generated TextGrids and, by doing so, significantly reduce the time required to create accurate TextGrids. Importantly, Autoscribe is open-source, easy to use, and readily accessible to individuals with little to no computer coding experience. Further, the tool adheres to ethical standards for data protection and participant confidentiality (e.g., HIPAA-compliance). In this paper, we start by explaining tool development. We then describe the methods and results of our tool validation process. This includes rigorously assessing the efficiency and accuracy of TextGrid creation for two conversational corpora with several differences (e.g., participant age, participant dialect, recording environment). Finally, we conclude with instructions for researchers and clinicians interested in applying and using the tool.

## Materials and methods

### Tool description

**Fig. 2:**
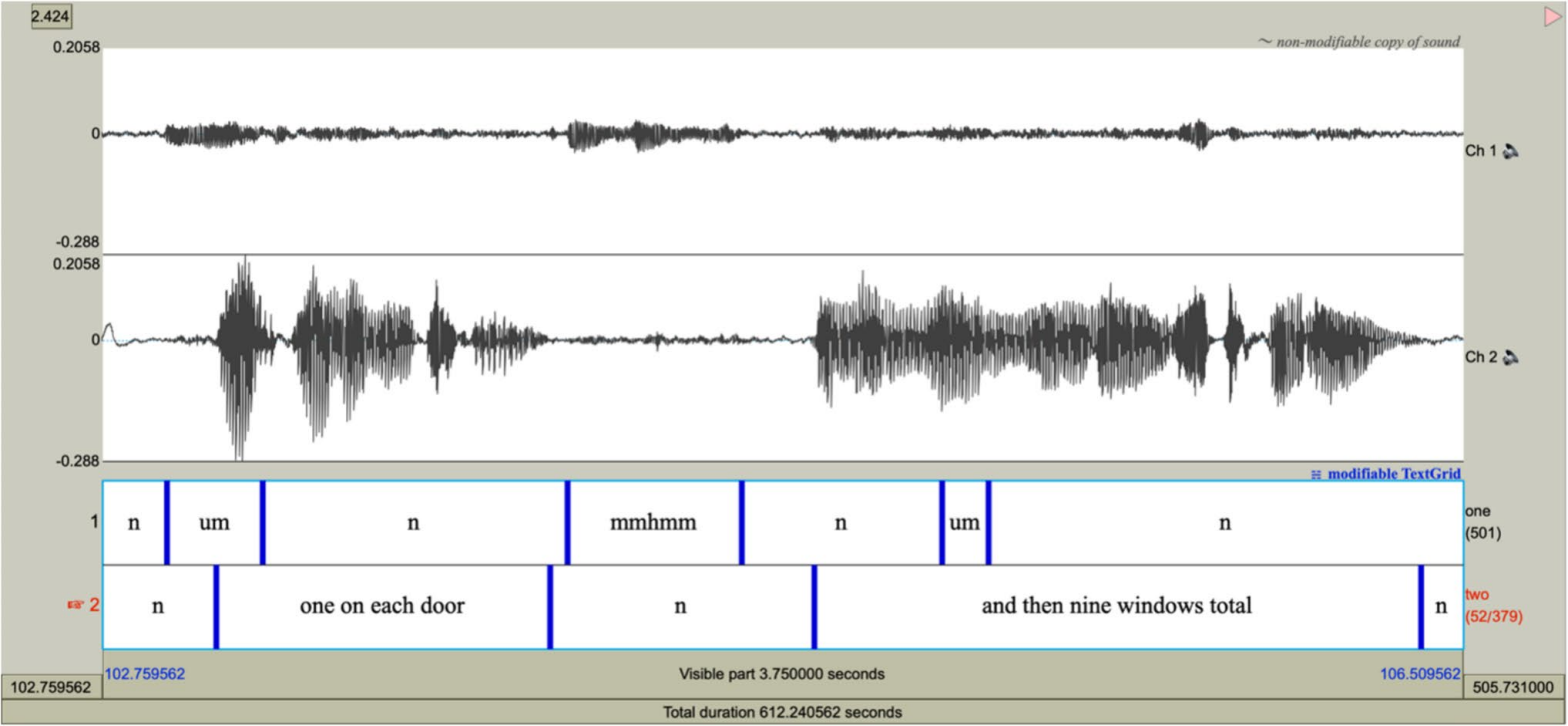
Example of completed Praat TextGrid. Each tier contains utterances segmented into transcribed speech and non-speech (represented with an “n”) for one speaker

Autoscribe was designed to take a two-channel .wav file of speech (one channel for each interlocutor) and produce a transcribed TextGrid. An example of a portion of a completed TextGrid is displayed in Fig. 2. The tool uses the R programming language, Python, and Praat together to prepare the file, assess speaking turns, split the .wav file into speaking turns, transcribe each turn, combine time stamps and transcription, and produce a TextGrid that can be imported into Praat. Conversational turns are delineated by marking boundaries between units of speech (with pauses no longer than .5 seconds, although this can be adjusted; see Table 1, *minimum silent interval* option) and non-speech. Within each boundary, all speech is transcribed word for word, and segments of non-speech are delineated with a lowercase “n” (although the specific string delineating non-speech can be controlled by the end user).

**Table 1:** Options under the control of the user of Autoscribe, including the default and alternatives

Option name	Description	Default	Alternatives
Remove Partial	Whether to keep words that are incomplete in the transcript	False	True
Hyphen	Whether hyphens be retained or replaced. There are three options: keep all hyphens (keep), remove all hyphens with no space where a hyphen was (remove), or remove all hyphens and insert a space where the hyphen was (space).	Keep	Space, Remove
Remove Apostrophe	Whether apostrophes should be removed	False	True
Remove Punctuation	Whether all other punctuation (i.e., besides apostrophes and hyphens) should be removed	False	True
Lowercase	Whether all uppercase text should be converted to lowercase	False	True
Non-speech	The string used to delineate non-speech segments	n	Any string of letters and numbers
Model Type	The model size to be used	Base	Tiny, small, medium, large, large-v2, large-v3
Prompt	The prompt that influences how Whisper model transcribes. Without a prompt similar to this, Whisper may not transcribe fillers and disfluencies	“I was like, was like, I'm like, um, ah, huh, and so, so um, uh, and um, mm-hmm, like um, so like, like it's, it's like, i mean, yeah, uh-huh, hmm, right, ok so, uh so, so uh, yeah so, you know, it's uh, uh and, and uh.”	Any textual prompt, but recommended to use fillers and disfluencies if present in the .wav file
Noise Reduction	Background noise reduction via Praat; can be useful for improving textgrid quality for conversations recorded in noisy environments	False	True
Silence Threshold	The silence threshold (relative to maximum intensity) in dB for determining non-speech segments	−45 dB	Any negative real number
Minimum Silent Interval	Minimum interval of silence to be considered as non-speech segment	0.5 seconds	Any positive real number
Minimum Sound Interval	Minimum interval of speech to be considered speech segment	0.1 seconds	Any positive real number
Minimum Pitch	Minimum pitch in the turn boundaries calculation in Praat (for advanced users)	100 Hz	Any integer
Time Step	Time step parameter in the turn boundaries calculation in Praat (for advanced users)	0 seconds	Any real number

*Note*. More details regarding Praat parameters can be viewed at https://www.fon.hum.uva.nl/praat/

The transcription uses OpenAI’s Whisper model, a speech processing model trained on over 680,000 hours of multilingual audio. The model, openly released by OpenAI, is best at transcribing English but also has performed well for other languages such as Spanish and German. There are several options to use different size models (e.g., base, small, medium, large); transcription accuracy tends to increase with larger models but computation time also tends to increase with larger sizes. By default, Autoscribe uses a custom prompt that is designed to help Whisper transcribe filler words and disfluencies. Importantly, like many features, the custom prompt is adjustable by the user to fit the needs of each situation. Beyond the prompt, Autoscribe has several other options available depending on the user’s needs. Table 1 highlights these options. Additionally, a description of when certain options may be most appropriate is included in the manual at https://humaninteractionlab.com/tools/autoscribemanual.

Importantly, because all calculations happen locally (i.e., are not calculated on a server but rather on the user’s own computer), the tool is compliant with data privacy and participant confidentiality standards. No data are shared with outside entities. That does mean, however, that the specifications of the user’s computer do determine the speed and possible sizes of the models available to be run.

### Tool validation

Autoscribe’s performance was evaluated using two conversational corpora. These conversational corpora were selected because they contrast each other in a number of ways (see Table 2). Further details regarding each corpus are described below.

**Table 2:** Characteristics of conversational corpora

Corpus	Corpus One	Corpus Two
Interlocutor age	Adults (age 46–84 years)	Children and adolescents (age 9–14 years)
Interlocutor dialect	Speakers of General American English	Speakers of Southern British English
Location	Conversations recorded in Utah, USA	Conversations recorded in London, UK
Conversation type	Rapport-building and task-based conversations	Task-based conversations
Recording environment	Conversations collected in various naturalistic settings, such as research laboratories, participants’ homes, public libraries, or community centers; interlocutors in the same room as one another	Conversations collected in a highly controlled laboratory setting; interlocutors in different rooms from each other
Recording quality	Good recording quality with some ambient noise and minimal microphone spill between interlocutors	Excellent recording quality with minimal ambient noise and no microphone spill between interlocutors
Microphone	Shure SM35 Performance Headset Condenser Microphone	Beyerdynamic DT297

#### Conversational corpora

##### Corpus #1

Our first corpus included 28 conversations collected by researchers at Utah State University^1^. This corpus includes both task-based and rapport-building conversations between typically communicating adult interlocutors between 46 and 84 years of age. All speakers were native speakers of General American English. Task-based conversations were elicited using the Diapix Task (Baker & Hazan, 2011), a collaborative spot-the-difference task where conversational dyads work together to identify 12 differences between sets of pictures. Rapport-building conversations were elicited using an adapted version of the Relationship Closeness Induction Task (Sedikides et al., 1999). In this task, participants are given a list of questions which they are instructed to discuss with their conversation partner. All conversations for this corpus were recorded using Shure SM35 performance headset condenser microphones and Zoom H4N recorders. Conversations were conducted in various quiet locations, including research laboratories, participants’ homes, public libraries, or community centers. Conversation partners engaged in the conversation while sitting face-to-face approximately six feet from one another. All conversations lasted 10 min.

##### Corpus #2

Our second corpus included 30 randomly selected conversations from audio recordings collected and made available to the research community by Hazan et al. (2016; see also Bradlow n.d.) at University College London (UCL). All interlocutors were typically communicating children and adolescents between the ages of 9 and 14 years and all were native speakers of Southern British English. In this corpus, task-based conversations were elicited using the Diapix Task. During conversations, interlocutors sat in different rooms and communicated via headset Beyerdynamic DT297 microphones. Conversations were recorded using E-MU 0404 USB audio interface (E-MU; Dublin, Ireland) and Adobe Audition software. Conversation length for the 30 conversations varied between 4 and 11 min (*M* = 8.45, *SD* = 1.88).

### Procedure

The majority of the default options of Autoscribe were selected to produce the TextGrids for the validation (see Table 1). However, a number of modifications were adopted:

1. For the first corpus, varying threshold values were used, ranging from – 40 dB (where channels had little to no overlap in noise) to – 25 dB (some overlap is apparent, e.g., speaker on channel 1 can be heard clearly on channel 2). The second corpus had no overlap between the channels and therefore used – 45 dB for all recordings.
2. Noise reduction was used in an effort to reduce the influence of noises (e.g., background spill from other channels, external noises in the environment) influencing the transcription.
3. We selected the “small” Whisper model, a somewhat larger model than the “base” default.
4. We also removed punctuation, replaced all hyphens with spaces, and removed any partial words from the transcript.

For running this in the R programming language, the general syntax was used:

- auto_textgrid(
- file, noise_reduction = TRUE, threshold = −30,
- model_type = "small", remove_punct = TRUE,
- hyphen = "space", remove_partial = TRUE)

### Data analysis

#### Efficiency

Following the creation of TextGrids, trained research assistants hand-checked each of them, correcting words that were transcribed incorrectly and adjusting inaccurate TextGrid boundaries. During this process, research assistants recorded the time (in minutes) it took to check and correct each TextGrid. Research assistants also annotated (i.e., created boundaries between speech and non-speech segments) and transcribed 28 TextGrids from the first corpus and 30 TextGrids^2^ from the second corpus entirely by hand and recorded the amount of time it took to complete this process. The efficiency of Autoscribe was then determined by calculating the percentage of time saved by using Autoscribe in comparison to manual TextGrid creation.

#### Accuracy

To determine the accuracy of the automated tool, we compared the TextGrids created by Autoscribe (i.e., the original uncorrected TextGrids) to those that had been checked and corrected by research assistants. We then assessed both transcription accuracy and boundary placement accuracy. Transcription accuracy was determined using three approaches: analysis of word error rate (WER), percent of words correct in the uncorrected transcripts using Autoscore (Borrie et al., 2019), and similarity via cosine distance. WER was calculated as the sum of substitutions, deletions, and insertions divided by the total number of words. For similarity, we used a metric known as cosine distance between q-gram profiles (Pikies & Ali, 2019; van der Loo, 2014) to quantify differences between corrected and uncorrected TextGrids. This metric ranges from 0 (perfect match) to 1 (completely different). We then report the most common words that differed from the uncorrected to the corrected TextGrids. Throughout the analyses, if words were transcribed on the wrong interlocutor’s tier (e.g., due to microphone bleed), these words were counted as incorrect. Accuracy of utterance boundary alignment was determined by calculating the proportion of boundaries in the uncorrected and corrected TextGrids that were within 0.10, 0.20, and 0.50 seconds of each other.

#### Reproducibility

To determine the reproducibility of TextGrids using this tool, we reran a subsample of ten transcripts. We then analyzed the percentage of the transcription and boundaries that are identical between the original and new TextGrids.

Statistical analyses were conducted in R version 4.5.1 with the tidyverse (Wickham et al., 2019), stringdist (van der Loo, 2014), readTextGrid (Mahr, 2024), data.table (Barrett et al., 2024), gtsummary (Sjoberg et al., 2021), wersim (Proksch et al., 2019, quanteda (Benoit et al., 2018, and tidytext (Silge & Robinson, 2016) packages. Code, output of the analyses, and an example of a completed textgrid, .txt file, and .csv file are provided at https://osf.io/sqh52.

## Results

### Efficiency

For the first conversational corpus, manual TextGrid creation took an average of 133 min per TextGrid (SD = 19 min, range = 102–205 min). Contrastingly, correcting automated TextGrids took an average of 38 min per TextGrid (SD = 10 min, range = 30–78 min). For the second conversational corpus, manual TextGrid creation took an average of 156 min per TextGrid (SD = 67 min, range = 58–401 min). Contrastingly, corrected automated TextGrids took an average of 45 min per TextGrid (SD = 15 min, range = 24–89 min). Thus, for both corpora, using the automated tool decreased the time needed to create a TextGrid by an average of 71%.

### Accuracy

#### Transcription accuracy

**Fig. 3:**
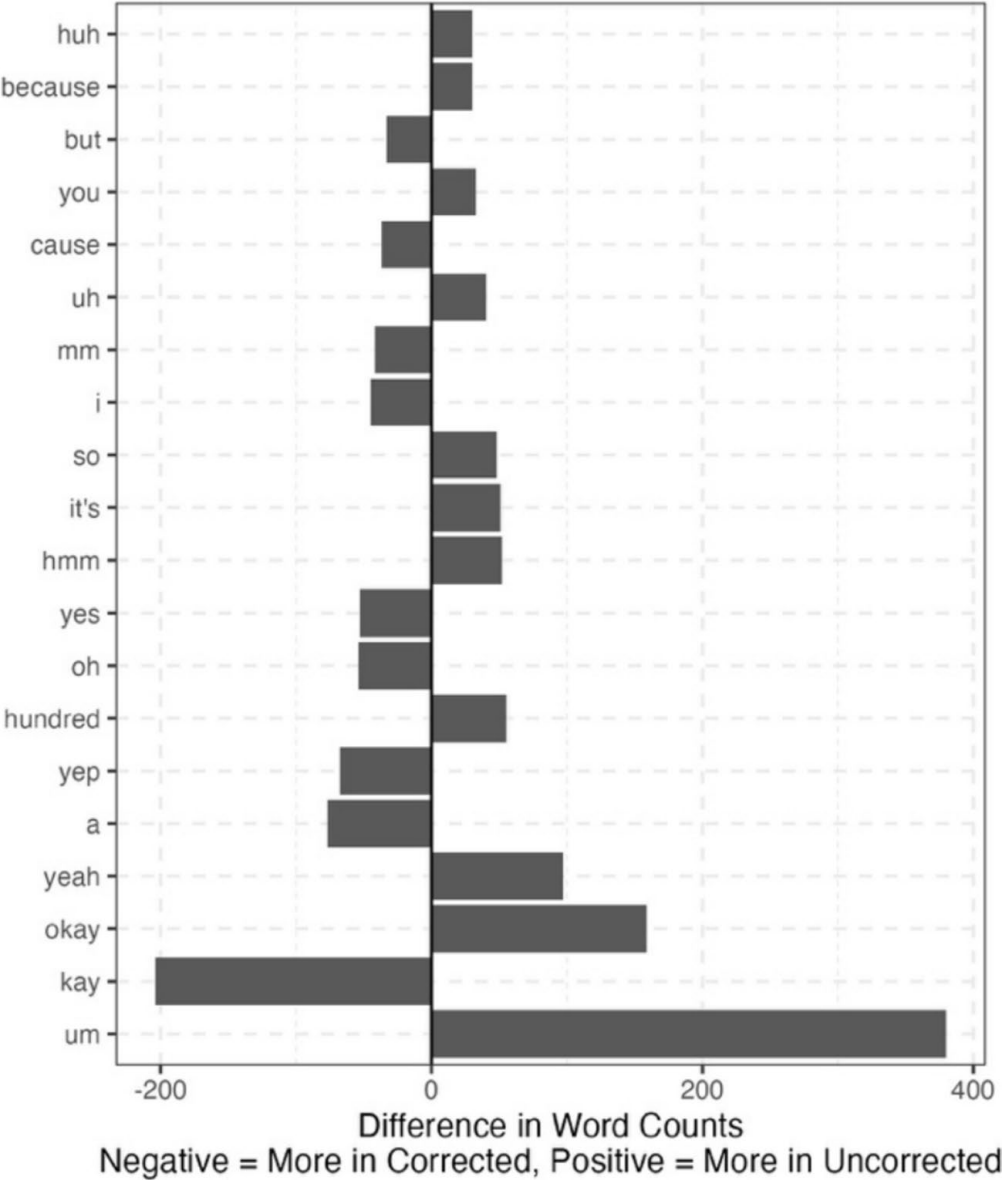
Word frequency differences between the corrected and uncorrected transcripts for the first corpus

**Fig. 4:**
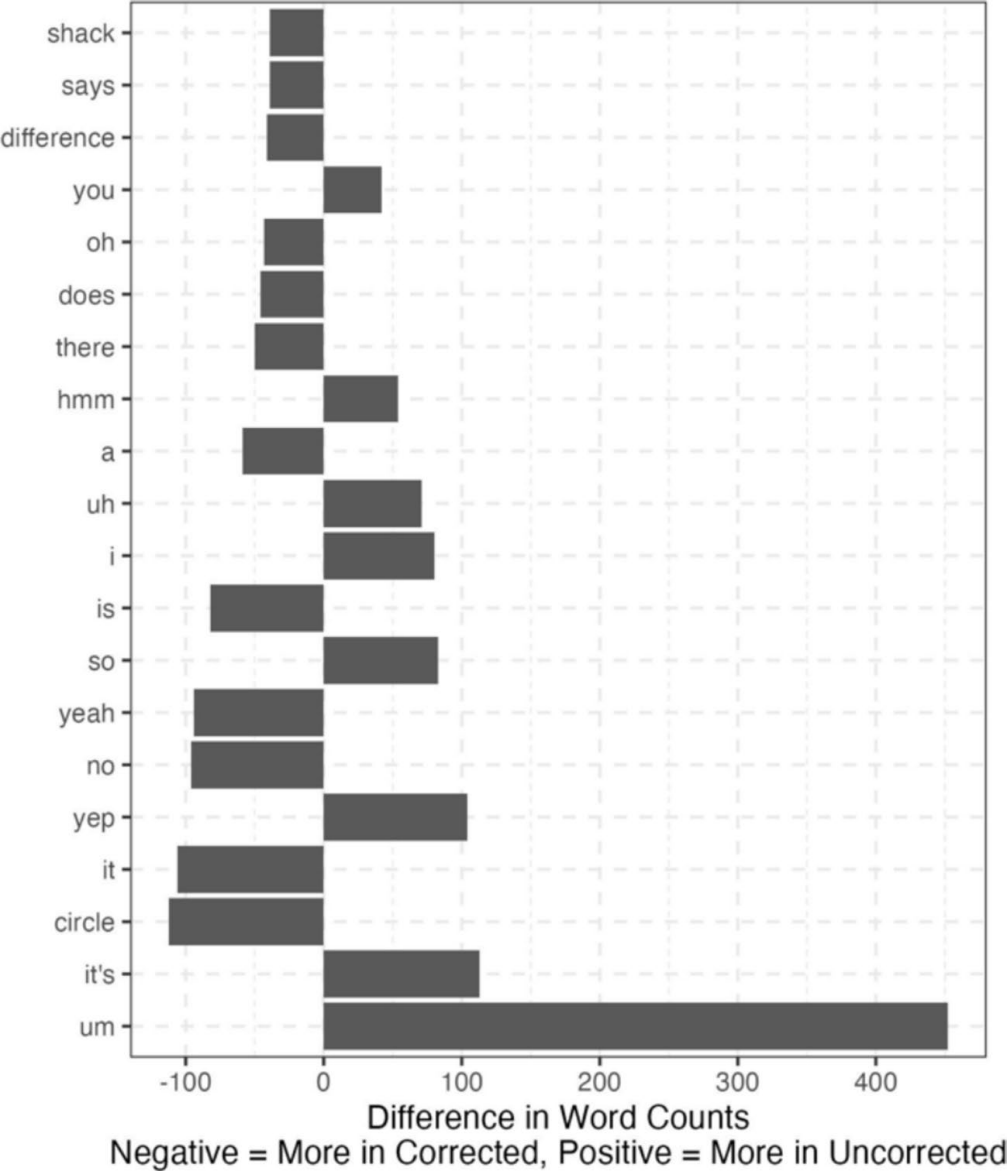
Word frequency differences between the corrected and uncorrected transcripts for the second corpus

The average WER per conversation was 0.16 (SD = 0.11) for the first corpus and 0.18 (SD = 0.10) for the second. In the first corpus, 60% of errors were substitutions, 6% were insertions, and 34% were deletions. In the second corpus, 74% of errors were substitutions, 8% were insertions, and 18% were deletions. The average percentage of words correct per conversation was 92% (SD = 4%) for the first corpus and 89% (SD = 5%) for the second corpus. The cosine distance was 0.0004 on average (SD = 0.0006) for the first corpus and 0.0008 on average (SD = 0.0009) for the second corpus. Both of these values are close to 0 with very little variability, signifying close alignment between the uncorrected and corrected transcripts. The most common word differences for the first corpus are shown in Fig. 3. This highlights the current extra use of “um” for the tool and how often it misses “kay.” These two words account for 584 of the total 4325 frequency differences in this corpus. The most common word differences are shown in Fig. 4. As before, this shows the current extra use of “um” for the tool, and that it uses “it’s” in place of “it is.”

#### Boundary placement accuracy

**Fig. 5:**
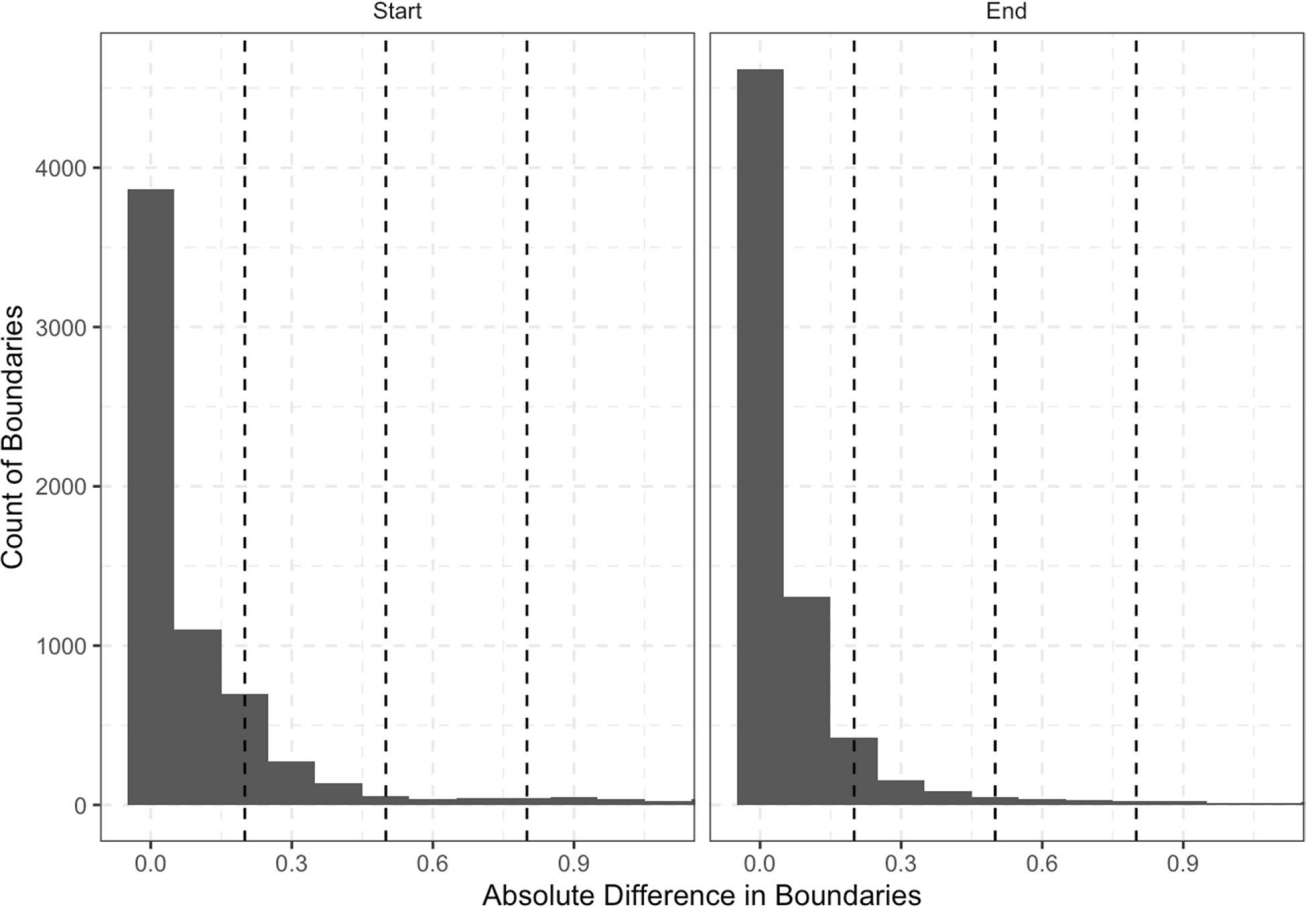
Absolute difference in boundaries between uncorrected and corrected TextGrids for the first corpus

For the first corpus, 86% of the boundaries were correctly identified (i.e., 14% of boundaries were extraneous and needed to be removed). Of the correctly identified boundaries, the median values of 96% of initial boundaries (interquartile range = 93% to 98%) and 89% of final boundaries (interquartile range = 85% to 94%) fell within 0.50 s of the corrected boundary (see Fig. 5). This included 91% of initial boundaries and 79% of final boundaries that fell within 0.20 s of the corrected boundary, and 83% of initial boundaries and 69% of final boundaries that fell within 0.10 s of the corrected boundary.

**Fig. 6:**
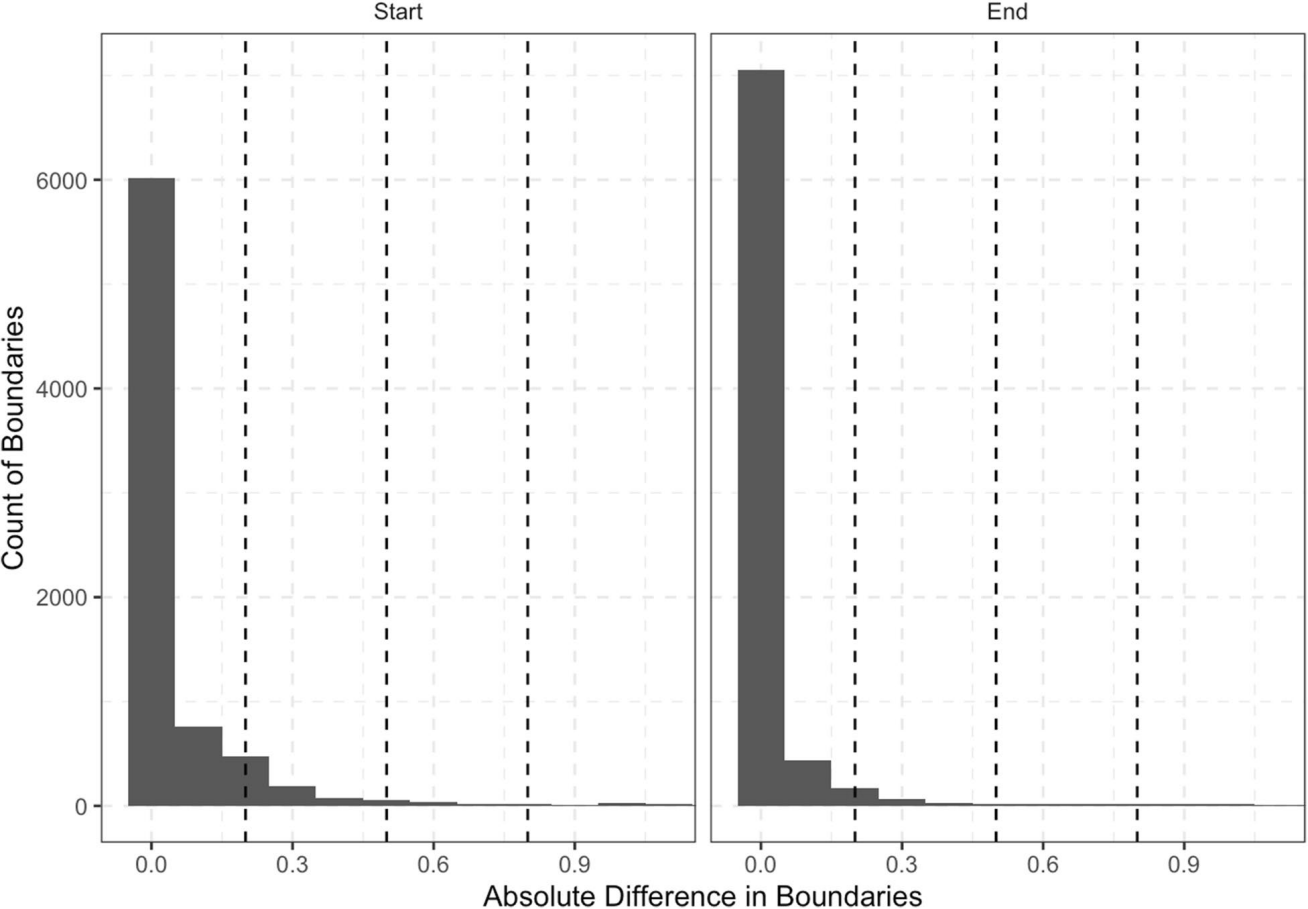
Absolute difference in boundaries between uncorrected and corrected TextGrids for the second corpus

For the second corpus, 99% of the boundaries were correctly identified (i.e., 1% of boundaries were extraneous and needed to be removed). Of the correctly identified boundaries, the median values of 98% of initial boundaries (interquartile range = 96–99%) and 96% of final boundaries (interquartile range = 93–98%) fell within 0.50 s of the corrected boundary in uncorrected and corrected TextGrids (see Fig. 6). This included 96% of initial boundaries and 90% of final boundaries that fell within 0.20 s of the corrected boundary and 93% of initial boundaries and 83% of final boundaries that fell within 0.10 s of the corrected boundary.

#### Reproducibility

Test–retest reliability indicated that boundaries were identical in 100% of instance. Transcripts were 91% identical from one run to another, with the majority of differences being insertions/deletions. This expected behavior is due to the design of the underlying Whisper model, and can result from using different prefixes and suffixes, different spellings of words, among other reasons.

## Discussion

The purpose of this project was to develop a tool to create Praat TextGrids from conversational audio recordings and validate this tool’s performance in terms of accuracy and efficiency. In doing so, our aim was to decrease the time and labor needed to prepare conversational data for further analysis. Our validation process was completed on conversations from two corpora that varied from one another on a number of features. Evidence of tool validity was apparent across both conversational corpora.

### Efficiency

From the onset of this project, we knew that, at this point, achieving accuracy at levels equal to those achieved by humans was unrealistic and that TextGrids would need to be checked and manually corrected after their initial creation. Accordingly, our ultimate goal was to create a tool for which the checking process was substantially faster than manual TextGrid creation. The attainment of this goal is noted in the fact that the amount of active working time needed for TextGrid creation decreased by over 70% for both corpora when using the automated tool. To put this into perspective, the amount of time saved on TextGrid creation for these 58 conversations was nearly 100 human hours. Here we note while our protocol for manual correction was extensive, with research assistants focusing on precise adjustments to transcription accuracy and boundary alignment, the degree of manual corrected needed will depend on the reasons for using the tool and the type of analyses being conducted. For example, while some types of analyses require extremely precise boundary alignment, others do not. Therefore, in some instances, the time required to correct TextGrids will be even faster than reported here.

### Accuracy

The accuracy of transcriptions was high. In our first corpus, which included adult interlocutors conversing in General American English, words were transcribed with 92% accuracy (broadly defined as percent words correct). In our second corpus, which included children and adolescents conversing in Southern British English, transcription accuracy was 89%. It is notable that Autoscribe is able to capture children/adolescent speech from this corpus with such a high degree of accuracy. Additionally, the versatility of this tool is reflected in the high accuracy achieved across two different dialects. Further, through fine-tuning, we were able to achieve this level of transcription accuracy in conversational speech that includes frequent repetitions, filler words, back-channeling, and interruptions.

The accuracy of utterance boundary placement was also high. In our second corpus, in which conversations were recorded in a high-quality recording environment, boundaries were correctly identified in 99% of instances and accurately placed (within 0.5 s) approximately 97% of the time. The accuracy of boundaries for our first corpus was slightly lower (i.e., correctly identified 86% of the time and accurately placed approximately 93% of the time), likely due to this corpus including conversations recorded in less ideal environments. Thus, while high-quality microphones and recording equipment were used, background noise impeded boundary alignment accuracy. Further, because participants were in the same room as one another, microphone spill between interlocutors led to some of these inaccuracies. Still, despite the obstacles that come with more ecologically valid recording environments, accuracy was still high, indicating that this tool can be used in many different types of settings.

### Application

Autoscribe is available in two different formats. The simplest option is the desktop application (currently available for macOS with Apple Silicon) version of the tool available at http://www.humaninteractionlab.com/tools. On this website, a manual is provided, which offers step-by-step instructions for downloading and using this tool. In addition, this manual provides more details about researcher options, suggestions for improving tool accuracy, and troubleshooting guidelines. The tool can be installed using the standard approach on macOS. Once installed, the first use of the tool will require additional setup that occurs automatically (i.e., sets up a distinct R instance and Python environment to keep the tool separate from the user's R program and any Python environments already on their machine). This will take a few minutes to set up, but after the first use, the tool will start up quickly. The application is then able to take a single .wav file or multiple .wav files and process them. At the end of the processing, a TextGrid (for an individual .wav file) or a zip folder (for multiple .wav files) will be available in the folder the user selects.

For individuals with experience with R, this tool can also be downloaded as an R package from GitHub at https://github.com/Human-Interaction-Lab/wav2textgrid. The main function is auto_textgrid(), with the main arguments shown in the example code in the Methods section. Several other arguments exist that can be reviewed in the help menu in R. If using the R package version of this tool, there are a number of package dependencies, including reticulate (to communicate between R and Python), the tidyverse, furniture, readtextgrid, English, some formatting tools (e.g., cli, scales) and several audio processing tools (e.g., seewave, speakr, tuner) (Ushey et al., 2025; Wickham et al., 2019; Barrett & Brignone, 2017; Mahr, 2024; Fox et al., 2021; Csárdi, 2025; Wickham et al., 2025 Sueur et al., 2008; Coretta, 2024; Ligges et al., 2023; Hester et al., 2025; Hester & Bryan, 2024). To run the code as an R package, it is recommended to set up a Python environment with the aid of reticulate for the Whisper model to download and run (see https://github.com/Human-Interaction-Lab/wav2textgrid/blob/master/README.md). All dependencies will be auto-downloaded (either as part of the package installation or at runtime). As a note, temporary files will appear in the folder where the .wav file is located during TextGrid creation. These will be deleted at the end of the calculation but are a necessary part of the process. The finished product is the file with the same name as the .wav file with “_output.TextGrid” appended and can be opened in Praat as well as any text editing programs.

Whether using the desktop application or the R package, the tool will provide notifications throughout the processing to inform users of progress. Regardless of format, the main computational load is the use of the Whisper models. It is important to ensure that the computer used to run Autoscribe meets the recommendations. For the small model (the one used throughout this report), having 4GB of RAM and 4–8 CPU cores is sufficient. For the base model, 2–3GB of RAM and 4 CPU cores are sufficient. For larger models (medium, large), more than 8GB of RAM may be required and more than eight CPU cores.

The utility of Praat TextGrids means that data generated from Autoscribe can be used to answer many types of research questions. Within Praat, additional features can also increase the utility of the TextGrids created by Autoscribe. For instance, additional tiers can also be added to the generated TextGrid to mark specific phonetic, morphosyntactic, or pragmatic elements of the conversation for further analysis. Even for researchers unfamiliar with Praat or whose analyses rely on other types of software, the TextGrids provided by Autoscribe can serve useful purposes. Information generated from Autoscribe, such as the tier name, transcript content, and timestamps of each utterance, can be converted into several different file types, including .csv or .txt files that can then be used for subsequent analyses. Data from Autoscribe can also be integrated with other software and tools, such as Python, R (R Development Core Team, 2024), ELAN (ELAN, 2024), and MATLAB (The MathWorks Inc., 2024) for further processing. In our research, Praat TextGrids (similar to those derived from Autoscore) have been used to explore conversational alignment in both acoustic (e.g., Wynn et al., 2022, 2023) and linguistic (Chieng et al., 2024) modalities; investigate acoustic features of speech in different types of speakers (e.g., Borrie et al., 2022; Wynn et al., 2022), and contexts (Wynn et al., 2024; Borrie et al., 2020); and share data from conversational corpora with the research community (Eijk et al., 2022). TextGrids are not limited to these purposes. Other researchers may find Autoscribe helpful for generating TextGrids to look at turn-taking dynamics, the use of back-channelling, the interplay between different communication modalities, code-switching patterns, dialogue structure, and linguistic patterns that occur in conversation.

## Limitations

While Autoscribe offers a promising avenue for advancing conversational research, there are some conversational contexts in which this tool’s efficiency is currently unknown. We note that the corpora used for tool validation represented conversations that included interlocutors from a wide age range and two different dialects. However, all speakers were at least nine years of age. As automatic speech recognition tools are generally trained on adults, tool performance on conversations involving younger children (whose lexical, syntactic, and articulatory patterns that differ from adults), may be less successful.

Additionally, all speakers in our corpora were typically communicating individuals. Informal exploration of the tool’s use in conversations of speakers with mild to moderate dysarthria has shown good results. However, the tool’s performance on individuals with more severe and/or different types of speech disorders is unknown. All validated conversations were also conducted in English. In theory, this tool can be used for conversations involving other languages. Indeed, in an informal exploration of this tool’s use in (relatively low-quality) audio recordings of conversations between Dutch speakers (with mild dysarthria), the authors found that the tool did work effectively (although the tool was somewhat less accurate than with English conversations, sometimes transcribing speech in other languages). However, the accuracy of transcripts will likely depend on the language and dialect being used. As new models become available, we intend to incorporate higher-performing open models into this tool.

In addition to speaker characteristics, this tool may also be impacted by audio quality. It is important to note that this tool can only be used in dyadic conversations when individual interlocutors are recorded on separate channels. Additionally, while this tool was validated on conversations that took place in various settings, the recording equipment used for all conversations was of high quality, and strategies were implemented to optimize audio quality as much as possible. As mentioned above, informal exploration of this tool’s performance did show relatively high accuracy with low-quality recordings. However, the degree to which audio quality impacts performance accuracy is unknown.

Finally, we note that transcripts produced by this tool use a specific transcription model (i.e., OpenAI’s Whisper model) and as such it relies on the accuracy and training data of that model. Research has demonstrated these tools to be adequate for social science research (Naffah et al., 2025), and given the current pace of development in artificial intelligence and transcription, it is likely that this will only improve with time. The tool was developed to be able to use updated models from OpenAI (or other available models) as they become available and may, in future iterations, incorporate other available models (e.g., Wav2Vec). Of particular note will be whether these models can be developed to better incorporate speech disorders or other inclusive contexts that will provide additional ability for accurate transcription.

## Conclusion

In sum, the purpose of this paper was to introduce Autoscribe, an open-source tool for creating Praat TextGrids from conversational audio recordings. We validated the tool in terms of accuracy, efficacy, and reliability on two conversational corpora that differed in many ways, allowing for exploration of tool applicability across conversations collected between different types of interlocutors and different recording environments. On both data sets, we demonstrated high levels of accuracy as well as a major advantage in terms of efficiency over traditional hand-created TextGrids carried out by trained research assistants. Thus, Autoscribe offers a user-friendly and versatile tool for conversational transcription that can be used for a variety of subsequent analyses.

## Data Availability

Code and output of the analyses is provided at https://osf.io/sqh52. This tool can also be downloaded as an R package from GitHub at https://github.com/Human-Interaction-Lab/wav2textgrid.

## Footnotes

1 The original corpus contained 30 conversations. However, two were removed due to poor audio quality.

2 Because the conversations in the second corpus were variable in length, time comparisons required the same audio files that were used to create textgrids using Autoscore to be used to create textgrids by hand. As all conversations in the first corpus were the same length, this was not necessary, and different audio files were used.
